# Nursing and the Disturbances of Breast Feeding

**Published:** 1915-09

**Authors:** Douglas P. Arnold

**Affiliations:** Buffalo. 758 Elmwood Avenue


### Nursing and the Disturbances of Breast Feeding.

DR. DOUGLAS P. ARNOLD, Buffalo.

Because of the comparative high mortality in artificially fed children and the fact that mother's milk is the natural, perfect, and most highly tolerated food, every infant under three months old is entitled and should receive breast milk. I say three months because the first three months of a child's life its food tolerance is not high and digestive upsets are most frequent and serious. Except in rare instances this nursing period can be lengthened with benefit to six or eight months when artificial feeding should supplement or take the place of mother's milk, for at this time the iron deposit in the child's liver is about exhausted and mother's milk no longer supplies the demand.

Breast feeding, like many other normal, every day events has been left to the mother, grandmother, friends and relatives to carry to a finish, and thanks to the babies' natural high tolerance, to a successful finish, and so we find nursing and its disturbances bound up in a tangle of illy founded facts and superstitions of which many physicians, I am sorry to say, more or less partake.

The first great question which we must consider is the possibility of nursing and we can now say with very rare exceptions that every mother can nurse her child, and that every breast milk, if sufficient in quantity is good. Every mother worthy of the name, who is made to understand the importance of breast feeding is willing and anxious to nurse her child.

Social conditions among the poor may make it difficult or impossible. These should be provided for. The contraindications are few. Tuberculosis of the mother being the chief one, here the infection of the child is due wholly to contact and not to milk infection.

Infections, per se, are not contraindications unless there is an attending cachexia or the mother's life is in danger. The child has a high grade of immunity until the sixth month and anti-toxins are supplied through the milk. Operations, menstruation, pregnancy, drug giving, etc., are not in themselves contraindications.

A syphilitic woman is duty bound to nurse her child, and, if given Salvarsan a curative effect is seen in the child's condition, but must never be considered a sufficient treatment. Cracked or painful nipples and even abscessed breasts can usually, with proper treatment, be remedied without resorting to weaning. Cracked nipples with the usual silver nitrate, compound tincture of benzoin, anesthesin and nipple shield. Abscessed breasts should be treated surgically. They are due to infection and milk stagnation; the nursing infant is the best preventive of caking, and seldom, if ever, receives enough pus through the plugged-up ducts to cause harm.

A month or so before the expected confinement, it is a good plan to see to it that the nipples protrude. The regular pulling out a couple of times a day helps, and the resulting mas sage toughens the nipples.

The regular flow of milk follows the colostrum the third or fourth day. This may be delayed until the third week and a good flow be finally established. Ordinarily a child is not placed on the breast the first twenty-four hours but may be supplied with water. A weak or premature child cannot stand starvation, so if the milk is slow in coming in, the child must be supplied with food, but must also be put to the breast regularly every three or four hours, this regular stimulation being necessary to excite the flow of milk. If the usual initial weight loss seems more than is warranted, supplementary feeding can be carried out with the same precautions, but care must be taken to stop this supplementary feeding as soon as the milk flow is established to prevent an overfeeding dyspepsia.

Due to the small fluid intake of the first few days, some children, with no apparent cause, react with a temperature of 105-106 F.; upon the addition of water or milk this is quickly dispelled and is probably due to salt concentration acting on the unstable heat center. It is, therefore, called Salt or Inanition Fever. This has no pathological importance but should not be confounded with infection.

A nursing mother can eat anything which does not disagree with her. The diet should be well balanced, nutritious, and contain two to four litres of fluid. The quality of the milk is not changed by feeding certain articles of diet except perhaps the increase of fat in the milk of poorly nourished women who are placed on a proper diet. The important and only milk stimulator is, (1) regular nursing, and (2) complete emptying of the breasts, the action of the so-called galactagogues being purely psychic. Other things being taken into account, the child should be nursed six times a day—at 10, 2, and 6, day and night, and a little later the two o'clock night feeding omitted. The mother should take a comfortable position, the nipple washed, and the nipple and part of the areola placed in the child's mouth. The suck reflex is the most primitive of all reflexes and most children easily develop the method of getting nourishment. A little patience is sometimes necessary, and the child's nose free for breathing. Idiocy must be thought of in a case which persistently refuses to nurse.

Premature or weak children must be fed expressed milk A Breck feeder is useful here.

A normal child can be allowed to nurse as long as it will—fifteen to twenty minutes being the average. Infants, like adults, differ in the rapidity with which they swallow their food. Thus a strong child on a full free breast may get enough in five minutes.

One half of the normal amount is usually taken the first five minutes, one fourth the second five minutes, and the remainder the rest of the time.

After a child has drunk enough it usually just plays with the nipple. The swallowing sounds are absent.

The disturbances of breast feeding are usually not as severe or dangerous as those of artificially fed children and are usually due to faulty technique, but when bad disturbances do occur, and in spite of proper feeding technique, the prognosis is not good, as it indicates a constitutionally poor child.

There are a few conditions which occur in thriving children which may seem disquieting, but if we are following the proper method of feeding we need not worry. I refer to spitting or vomiting and abnormal bowel movements. These conditions if aggravated and associated with stationary or loss of weight are distinctly pathological and will be discussed later.

''Spitting baby, healthy baby'' is an old adage which is often true. A strong, greedy child will eat too much, and too fast, and swallow a great deal of air with its food. An air bubble in the stomach is normal but too much causes regurgitation. If the old remedy of sitting the child up and patting it on the back is used, the air rises to the top of the stomach contents, and when expelled is less likely to bring food up with it. Other small devices, such as slow eating, placing child on right side and slightly raised, may be tried with good results. This atonic type of regurgitation cannot be confounded with the spastic vomiting of pyloric stenosis. Lavage once or twice daily and Novocaine .001 before each meal can be used with success in certain types of vomiting.

Constipation is quite common in breast-fed children, and is much to be preferred to diarrhoea. Children often go twenty-four hours without a stool. Enemas and medicine are to be avoided as much as possible. A fermentable sugar can be added in the form of a 10 per cent. malt extract, 20-30 c. c. to be given after meals, depending on the action on the bowels. After three months fruit juices can be given.

Instead of the so-called normal yellowish paste stools, some children will have greenish, watery, slimy stools, which often are increased in number, and still they gain in weight. Unless this is a premonitory symptom of dyspepsia (usually due to overfeeding) the stools can absolutely be disregarded. If the gain is not up to standard and the mother and doctor become nervous, ten to twenty grams of a milk casein preparation can be given (i. e., Plasmon, a powder, can be dissolved in two ounces or more of water, brought to a boil and, because it is not easily soluble, put through a fine sieve. About one-half ounce is given before nursing. This increases the alkalinity of the intestinal tract, counteracts the fermentation, slows the intestinal contents and makes the stools solid. If it is not readily taken it can be sweetened with saccharine.

We can divide the disturbances into

1. Dyspepsia (a) Overfeeding.
   (b) Parenteral Infection.
2. Undernourishment.
3. Disturbance of Balance.

**Dyspepsia.**

There is a condition the first few weeks of life called "Dyspepsia of the New-born." The child has a low tolerance for food, vomits and has diarrhoea. The chief danger here is the removal of the child, thinking that the breast milk is too strong. Such a child, showing dyspeptic symptoms on the highest tolerated of infant foods will certainly have trouble when put on artificial feeding. These symptoms are usually done away with when the child is placed on five feedings in twenty-four hours with possibly one-half to one ounce of barley gruel given before each feeding. If the child is then taking too much the nursing time can be limited. Alleitement mixte feeding should not be thought of before the third month.

Real dyspepsia is usually due to mistakes in feeding and is more common in this country where eight to ten feedings a day are often given. In this way a new feeding is placed on top of the previous one for it takes two or three hours for the stomach to empty itself and is should also have some time for self-disinfection. Finally the child's tolerance is overstepped. It vomits, has diarrhoea, loses weight and appetite and after several such attacks we may suddenly find that we are dealing with a mild case of atrophy; or the child may remain constipated and reach this same result through the inanition route. I will speak of this later.

The treatment consists in regular feeding five or six times a day and depending on the case, and the condition of the child, preceded by a 3-24 hour tea period, always remembering that these weak cases cannot stand starvation.

A too high fat content causing digestive disturbances is almost too rare to mention. If such a diagnosis is made it must be made from a twenty-four hour sample of milk, for milk varies from one-half to 10 per cent. depending on the time of day or time of nursing. This can be treated by using a diluent before feeding and in all cases of disturbance we must be certain that the water need of 150 c. c. per Kg. body weight is supplied. The nurse can be changed remembering that frequent nurse changing is always an indication of poor pediatrics.

When there has been no dietetic error and the child has been doing nicely, a sudden dyspepsia means infection. Every infection dyspepsia does not necessitate a tea period. It may be mild, cause no loss in weight, in fact, the child may continue to gain. Unless the dyspepsia is more severe the food is not changed and it clears up with the clearing up of the infection A child with a poor water binding power may lose a great deal of weight at the beginning of an infection but this is not the rule. It usually comes later with the secondary dyspepsia.

Overwarming and overcooling can cause dyspepsia.

Cathartics have no place in the infections of nursing infants as they may start a fatal diarrhoea.

If these milder disturbances of nutrition are not early or properly treated, the more severe nutritional disturbances can result but these are not as frequent or usually as severe as in artificially fed children.

**Undernourishment.**

Undernourishment on the breast is shown by stationary weight or gradual loss, bad tissue turgor, constipation, anemia, fluctuations of temperature and poor immunity. It usually occurs in the following way: a child is irregularly fed, only partially empties the breast; the milk therefore, due to lack of drainage, ''goes back,'' the child loses its appetite, loses weight, one or two bottles are added and the breast stimulation is taken away to a greater degree. The child is not getting its quota of 150 c. c. per Kg. body weight, and this can be told only by keeping account of the child's weight before and after nursing for twenty-four hours (single meals differ greatly,—from 40 to 300 c. c.) Now, possibly because the child is losing weight and does not seem to get enough milk it is given more meals a day, aggravating the present condition.

If the child is not taking enough milk per nursing and is on five meals a day, the number can be raised to eight, but when the child has been taking ten or twelve meals the number of meals must be reduced, care being taken that the already reduced child does not suffer from further inanition. This mistake is liable to happen when inanition is associated with diarrhoea, simulating dyspepsia; further starvation here would be fatal.

How do we treat the usual overfeeding dyspepsia?

If necessary, for vomiting, etc.—(1) A short tea period is given; then—(2) regular feedings, five to eight, depending on the causation of the trouble; (3) after each feeding the breasts are emptied either manually or with a pump. If the child's appetite is poor and the breasts are poor, trouble may be experienced in re-establishing the flow. There are then several possibilities: (1) 5 drops of essence of Pepsin can be given three times a day before meals to stimulate the appetite.

Then there is that large subject of "wet nursing." (1) Another child can be taken on, for breasts react to increased stimulus. From one to four children can stimulate a breast to secrete 3 to 4 litres, or, (2) The weak child can be placed on the milk rich breast and the strong child on the poor breasts and allow him to bring the secretion back. This is really unfair to the strong child, and care must also be taken that the weak child does not, because of poor emptying allow the good breasts to retrograde. It is much better to place the two children on the milk-rich breasts.

We all know that wet-nursing is fraught with social, moral and medical problems which are difficult to handle.

The age of the wet nurse and age of her child makes no difference as to quality of her milk, but we should never use a nurse whose child is under ten weeks, even with all our carefully conducted tests for syphilis. We can then also tell how well her child is thriving. We must never forget that we owe just as much protection to the wet nurse; and her child has the first moral right to its mother's milk.

Are these cases ever better off weaned?

Yes. After the third month in cases of so-called Disturbance of Balance with stationary weight, constipation, etc., coming on as described above or following an acute disease of some type. Alleitement mixte can be used with benefit.

These children can usually be classed under the headings of Neuropathia or Exudative Diathesis. They gain in weight remarkably if put on one or two feedings of a low fat, high carbohydrate food. So well do they gain that the mother must be cautioned not to wean the child entirely.

Because mothers' milk is low in albumen and salts, and these are the elements most needed for cell growth (the fastest growing animals being richest in these elements) mothers' milk is not a particularly good food for recovering a great weight deficit such as we find in the types of atrophy. In children with fluid stools a proteid preparation can be added (Plasmon). I spoke of this under "diarrhoea." Or, the child can be taken off breast milk and placed on Eiweissmilch, later to be transferred to ordinary milk mixtures.

There is one thought which I hope to have brought out in this paper, and that is that most mothers' milk is good, and many children are deprived of its benefit without sufficient cause and I hope to have given a few hints which will help to keep these children on their natural food.

758 Elmwood Avenue.

